# An investigational prognostic framework for stage IVB cervical cancer based on metastatic patterns: a population-based study with exploratory external evaluation

**DOI:** 10.3389/fonc.2026.1931638

**Published:** 2026-09-03

**Authors:** Bingbiao Lin, Xuehan Huang, Hongxin Huang, Keyan Xie, Jianzhou Chen, Chuangzhen Chen, Yizhou Zhan

**Affiliations:** 1 Department of Radiation Oncology, Cancer Hospital of Shantou University Medical College, Shantou, Guangdong, China; 2 Shantou Key Laboratory of Precision Diagnosis and Treatment in Women’s Cancer, Cancer Hospital of Shantou University Medical College, Shantou, China; 3 School of Medicine, Westlake University, Hangzhou, Zhejiang, China; 4 Affiliated Hangzhou First People’s Hospital, School of Medicine, Westlake University, Hangzhou, Zhejiang, China

**Keywords:** chemoradiotherapy, metastatic cervical cancer, metastatic pattern, population-based study, prognostic stratification

## Abstract

**Background:**

Current staging system classifies all patients with metastatic cervical cancer as stage IVB, without accounting for prognostic heterogeneity. This study aimed to develop an exploratory metastatic pattern-based framework for prognostic stratification within stage IVB cervical cancer.

**Methods:**

Patients with *de novo* stage IVB cervical cancer were identified from the Surveillance, Epidemiology, and End Results database and the Cancer Hospital of Shantou University Medical College. Random survival forest and Cox regression were used to identify key predictors of cancer-specific survival and develop a nomogram-derived scoring system for stratifying patients into IVB subgroups. Prognostic differences were evaluated using Kaplan-Meier and Cox regression analyses. Exploratory analyses further examined survival patterns according to treatment modality across subgroups.

**Results:**

Brain, liver, lung, and bone metastases were identified as the main metastatic determinants of prognosis. Based on their prognostic contributions and combinations, patients were categorized into three subgroups: stage IVB1 (metastases excluding brain, liver, lung, or bone), stage IVB2 (metastases excluding brain, including one of liver, lung, or bone, or combinations of liver and bone, or lung and bone), and stage IVB3 (metastases to the brain, or concurrent liver and lung). The three groups showed distinct survival patterns in the training and internal validation cohorts. In the external cohort, the overall prognostic ordering was similar; however, validation of the IVB3 category remained exploratory owing to its limited representation. In exploratory subgroup analyses, chemoradiotherapy was associated with longer survival than chemotherapy alone in stage IVB1 and stage IVB2, whereas no significant survival difference was observed between these approaches in stage IVB3.

**Conclusion:**

The proposed IVB1-IVB3 classification represents an investigational prognostic framework for characterizing heterogeneity within stage IVB cervical cancer. Treatment-related findings were observational and hypothesis-generating and should not be interpreted as evidence of treatment benefit. Prospective multicenter studies are required before this framework can be considered for clinical adoption.

## Introduction

Cervical cancer remains a major health concern for women worldwide. Because of its insidious onset, cervical cancer is often detected at an advanced stage; more than one-third of patients present with advanced disease, including approximately 13% with FIGO stage IVB disease, which is characterized by distant metastasis (1). Although the 5-year survival rate exceeds 90% for patients with early-stage or locally advanced cervical cancer, it decreases to less than 20% in patients with recurrent or metastatic disease (2).

Patients with stage IVB cervical cancer represent a clinically heterogeneous population, with substantial variation in survival despite being grouped within a single metastatic category (3, 4). Previous studies have shown that survival differs according to the involved metastatic organs and patterns of metastatic spread (5, 6). In several other malignancies, such as lung, colorectal, and nasopharyngeal cancers, patients with distant metastatic disease have been further subclassified to improve prognostic discrimination and support treatment stratification. By contrast, the current International Federation of Gynecology and Obstetrics (FIGO) classifies cervical cancer with distant organ metastasis uniformly as stage IVB, without accounting for differences in metastatic sites or combinations of metastatic involvement. This broad categorization may limit prognostic resolution and may not fully capture clinically relevant heterogeneity within stage IVB disease.

Advanced cervical cancer has traditionally been considered incurable, with an extremely poor prognosis. Systemic therapy, predominantly chemotherapy, remains the cornerstone treatment for recurrent or metastatic cervical cancer (7). Historically, local therapies targeting the primary tumor or metastatic sites were considered palliative, intended solely to alleviate symptoms. Nevertheless, emerging retrospective evidence indicates that selected patients might also derive benefit from locoregional treatments, such as radiotherapy or surgical intervention (8–10). In clinical practice, multimodal therapeutic approaches are frequently utilized with the purpose of prolonging survival and alleviating symptoms in advanced disease. However, the accumulation of toxicities and complications may yield counterproductive results.

Although previous studies have explored associations between different metastatic organs and prognosis in stage IVB cervical cancer, these studies largely rely on the number or broad patterns of metastatic sites and do not fully capture the prognostic heterogeneity of individual metastatic organs or their combinations (11, 12). A simple and clinically applicable system that refines stage IVB according to site-specific metastatic risk remains lacking. We therefore used the Surveillance, Epidemiology, and End Results (SEER) database and an independent institutional cohort to develop and evaluate an exploratory metastatic site-based prognostic framework within stage IVB cervical cancer. We further conducted exploratory observational analyses to examine whether associations between treatment modalities and survival differed across the resulting subgroups.

## Methods

### Patients

The SEER database collects comprehensive clinical data for retrospective analyses, including patient demographics, tumor details, diagnosis stage, treatment, and follow-up.

Patients diagnosed with *de novo* IVB stage cervical cancer (tumor site coded as C53.0–C53.1, C53.8, and C53.9) were identified during a study period of 2010 to 2021 using the SEER*Stat software (version 8.4.4) according to the International Classification of Diseases for Oncology, Third Edition (ICD-O-3). Inclusion criteria comprised age ≥ 18 years, a single primary tumor, and non-zero survival duration. Patients were excluded if data were missing on race, histology, grade, metastatic sites (brain, liver, lung, bone, distant lymph nodes, or other), treatments, survival time/status, or evidence of primary tumor. Since metastatic site data were recorded starting in 2010, this study included data from 2010 onward and recorded patients’ metastatic sites.

Data from 2018–2021 constituted the training cohort (N = 1171). For the 2010–2017 SEER cohort, the AJCC 7th edition classified para-aortic lymph node metastases as stage IVB but did not allow them to be reliably distinguished from other distant lymph node metastases within the available SEER data fields. To minimize potential contamination by cases that would be classified as stage IIIC under the contemporary FIGO 2018 criteria, patients with isolated distant lymph node metastasis were excluded from the pre-2018 cohort (13). The proposed IVB1 was therefore operationally defined based on identifiable visceral and/or other non-nodal distant metastatic sites. These data formed the internal validation cohort (N = 1,549), which was used as a limited temporal validation cohort rather than a full validation cohort encompassing all potential metastatic categories. The external validation cohort included patients treated at Cancer Hospital of Shantou University Medical College between January 2014 and February 2024 (N = 136), selected according to criteria used for the SEER cohorts. Follow-up data were obtained from the institutional follow-up office, telephone follow-up, and the hospital information system. Taxane-platinum chemotherapy was the most commonly used systemic regimen (N = 91). The most commonly prescribed primary-site radiotherapy regimens were 45/50 Gy in 25 fractions and 46 Gy in 23 fractions. All primary-site radiotherapy was delivered with curative intent, and metastatic-site radiotherapy was also administered with curative intent in the majority of patients receiving irradiation to both the primary and metastatic sites (52/55). The other-site metastases category includes heterogeneous metastatic sites not classified as liver, lung, bone, or brain, including the adrenal gland, bone marrow, pleura, malignant pleural effusion, peritoneum, and skin, as well as more generalized involvement such as carcinomatosis. In our external cohort, these sites included the pleura, adrenal gland, kidney, peritoneum, and ovary.

The primary and secondary endpoints in this study were cancer-specific and overall survival, respectively. Cancer-specific survival was defined as the interval from diagnosis to death attributable to cervical cancer or the last follow-up. Overall survival was defined as the interval from diagnosis to death from any cause or the last follow-up. Based on previous research, an age cutoff of 60 years was employed (14). To investigate the role of non-diagnostic surgery, this study only included patients who underwent radical or extended hysterectomy and pelvic exenteration.

### Statistical analysis

Descriptive statistics were used to summarize the distributions of demographic characteristics and clinical data. A random survival forest (RSF) model including 12 clinical variables was fitted in the training cohort to rank predictors of cancer-specific survival according to Variable Importance scores. Variables with higher Variable Importance values indicate greater predictor relevance. Univariate Cox regression and backward stepwise multivariable Cox regression were then performed. The Cox proportional hazards regression model was applied to calculate hazard ratios (HR) and 95% confidence intervals (CI) for each factor. Integrating results from both approaches, variables with high Variable Importance and statistical significance were retained for multivariate modeling, from which a nomogram was developed. Calibration of the nomogram was evaluated by comparing nomogram-predicted and Kaplan–Meier-estimated 3-year cancer-specific survival probabilities. Calibration curves were generated using 1,000 bootstrap resamples. The 45-degree diagonal line indicated perfect calibration. The metastatic-site scores were derived from the relative contributions of brain, liver, lung, and bone metastases in the Cox-based nomogram and rounded to clinically interpretable values of 10, 6, 6, and 3 points, respectively. The total score was calculated as 10 × brain + 6 × liver + 6 × lung + 3 × bone metastasis, with each site coded as present or absent. X-tile analysis in the training cohort identified optimal cutoffs of < 3, 3 - 9, and ≥ 10 points, defining low-, intermediate-, and high-risk groups, respectively, which were subsequently mapped to the IVB1–IVB3 substages.

The proportional hazards assumption was formally assessed for all Cox regression models using Schoenfeld residuals. Both variable-specific and global tests were examined. When the primary exposure variable showed evidence of non-proportionality, piecewise Cox sensitivity analysis was performed. For models with a significant global test but no evidence of non-proportionality for the primary IVB staging variable, the primary Cox estimates for IVB substage were retained.

Survival curves were generated using Kaplan-Meier (KM) methods, with log-rank tests assessing group differences; the effect of IVB precise staging on survival was evaluated via univariate and multivariate Cox regression. To mitigate potential immortal time bias in treatment-related survival comparisons, a 3-month landmark Kaplan–Meier sensitivity analysis was performed. Patients who died or were censored within 3 months after diagnosis were excluded, and survival time was recalculated from the landmark time point. The statistical analyses were performed using R 4.4.1. Statistics were considered significant for two-sided p-values < 0.05.

## Results

**Figure 1:**
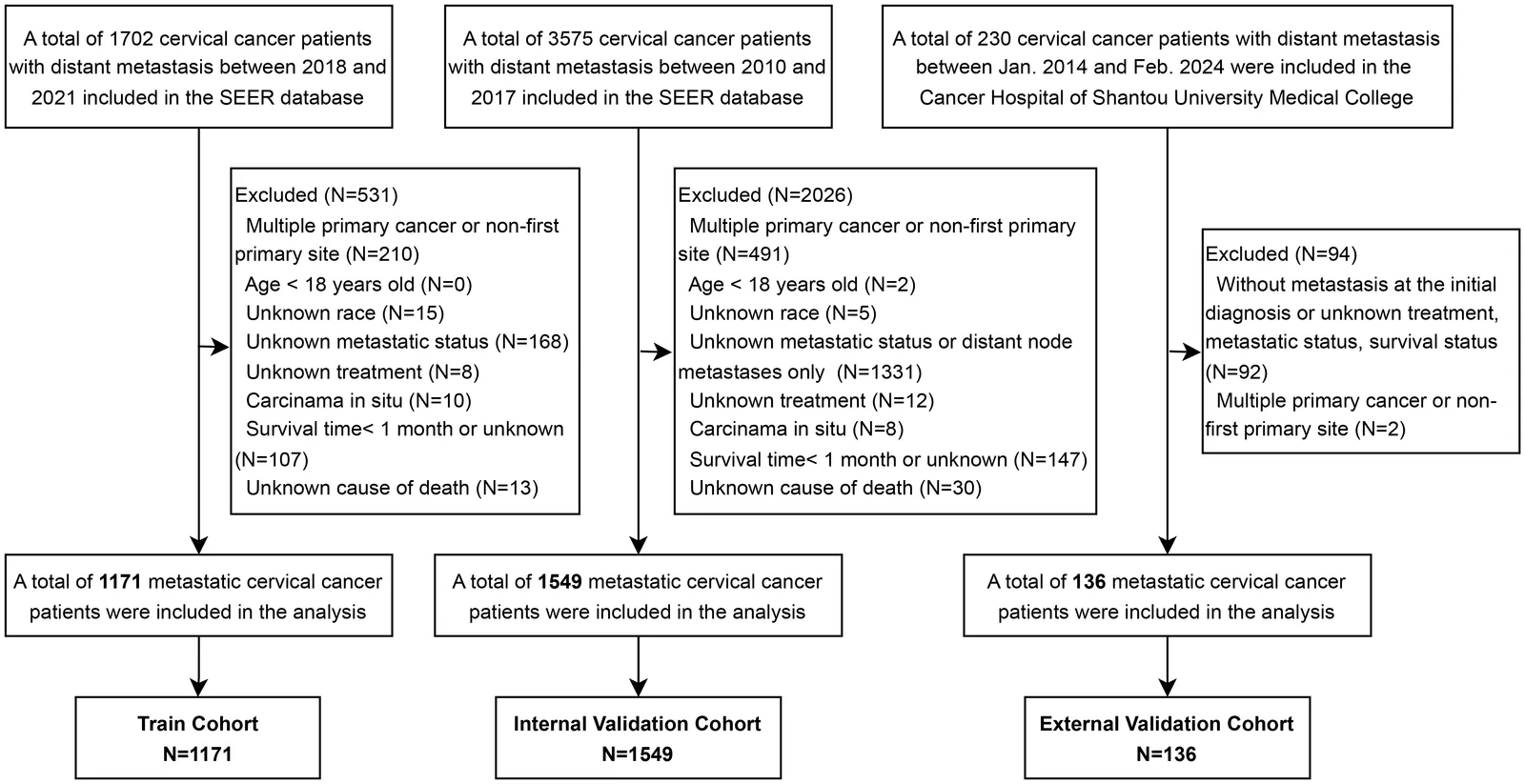
A flowchart of patient enrollment.

A total of 2856 patients were enrolled in this study (**Figure 1**), with comprehensive clinicopathological and therapeutic details summarized in **Table 1**. Distant lymph node metastases were the most frequent metastatic sites in the training cohort (51.1%) and the external validation cohort (69.9%). The lung (40.8%) was the most frequently involved organ for hematogenous metastases across all cohorts. Notably, because the AJCC 7th edition cannot differentiate para-aortic from distant lymph node metastasis, distant lymph node status was classified as ‘Unknown’ for all patients in the internal validation cohort. Relative to the SEER database, patients in the external validation cohort had higher rates of primary site surgery (16.9%), metastatic site surgery (14.7%), radiotherapy (69.9%), and chemotherapy (89.7%).

**Table 1:** Baseline patient characteristics.

Characteristics	Train cohort N=1171 (%)	Internal validation cohort N=1549 (%)	External validation cohort N=136 (%)	Total N=2856 (%)
Age, years				
< 60	687 (58.7)	921 (59.5)	100 (73.5)	1708 (59.8)
>= 60	484 (41.3)	628 (40.5)	36 (26.5)	1148 (40.2)
Race				
White	828 (70.7)	1132 (73.1)	0 (0.00)	1960 (68.6)
Black	212 (18.1)	263 (17.0)	0 (0.00)	475 (16.6)
Other	131 (11.2)	154 (9.94)	136 (100)	421 (14.7)
Histology				
SCC	716 (61.1)	880 (56.8)	114 (83.8)	1710 (59.9)
Non-SCC	455 (38.9)	669 (43.2)	22 (16.2)	1146 (40.1)
Histologic grade				
Grade I	30 (2.56)	43 (2.78)	0 (0.00)	73 (2.6)
Grade II	227 (19.4)	305 (19.7)	19 (14.0)	551 (19.3)
Grade III-IV	417 (35.6)	696 (44.9)	18 (13.2)	1131 (39.6)
Unknown	497 (42.4)	505 (32.6)	99 (72.8)	1101 (38.6)
T stage				
T1-2	362 (30.9)	466 (30.1)	77 (56.6)	905 (31.7)
T3-4	593 (50.6)	812 (52.4)	59 (43.4)	1464 (51.3)
Tx	216 (18.4)	271 (17.5)	0 (0.00)	487 (17.1)
N stage				
N0	248 (21.2)	451 (29.1)	20 (14.7)	719 (25.2)
N1-2	793 (67.7)	916 (59.1)	116 (85.3)	1825 (63.9)
Nx	130 (11.1)	182 (11.7)	0 (0.00)	312 (10.9)
Bone metastasis				
Yes	273 (23.3)	426 (27.5)	31 (22.8)	730 (25.6)
No	898 (76.7)	1123 (72.5)	105 (77.2)	2126 (74.4)
Brain metastasis				
Yes	34 (2.90)	53 (3.42)	0 (0.00)	87 (3.0)
No	1137 (97.1)	1496 (96.6)	136 (100)	2769 (97.0)
Liver metastasis				
Yes	230 (19.6)	360 (23.2)	13 (9.56)	603 (21.1)
No	941 (80.4)	1189 (76.8)	123 (90.4)	2253 (78.9)
Lung metastasis				
Yes	408 (34.8)	722 (46.6)	34 (25.0)	1164 (40.8)
No	763 (65.2)	827 (53.4)	102 (75.0)	1692 (59.2)
Distant lymph node metastasis				
Yes	598 (51.1)	0 (0.00)	95 (69.9)	693 (24.3)
No	573 (48.9)	0 (0.00)	41 (30.1)	614 (21.5)
Unknown	0 (0.00)	1549 (100)	0 (0.00)	1549 (54.2)
Distant other sites metastasis				
Yes	412 (35.2)	472 (30.5)	6 (4.41)	890 (31.2)
No	759 (64.8)	1077 (69.5)	130 (95.6)	1966 (68.8)
Surgery to primary site				
Yes	93 (7.94)	175 (11.3)	23 (16.9)	291 (10.2)
No	1078 (92.1)	1374 (88.7)	113 (83.1)	2565 (89.8)
Surgery to distant sites				
Yes	89 (7.60)	152 (9.81)	20 (14.7)	261 (9.1)
No	1082 (92.4)	1397 (90.2)	116 (85.3)	2595 (90.9)
Radiation				
Yes	635 (54.2)	927 (59.8)	95 (69.9)	1657 (58.0)
No/Unknown	536 (45.8)	622 (40.2)	41 (30.1)	1199 (42.0)
Chemotherapy				
Yes	904 (77.2)	1133 (73.1)	122 (89.7)	2159 (75.6)
No/Unknown	267 (22.8)	416 (26.9)	14 (10.3)	697 (24.4)

N, number.

The training cohort comprised 1171 patients, with a median follow-up of 8 months (IQR: 3–16 months). The 1-year and 3-year overall survival rates were 48.6% and 22.4%, respectively. The internal validation cohort included 1549 patients, with a median follow-up of 9 months (IQR: 4–21 months), and corresponding 1-year and 3-year overall survival rates of 40.1% and 16.5%. In the external validation cohort of 136 patients, the median follow-up was 25 months (IQR: 12–59 months), with 1-year and 3-year overall survival rates of 80.0% and 50.7%.

Utilizing a RSF algorithm, the variables with the highest prognostic impact in the training cohort were lung metastasis, brain metastasis, liver metastasis, and bone metastasis (**Supplementary Figure 1**). Subsequent multivariate Cox proportional hazards regression with backward stepwise selection confirmed independent associations with cancer-specific survival for older age, Tx staging, bone metastasis, brain metastasis, liver metastasis, and lung metastasis (**Supplementary Table 1**). Based on the overlap between variable-importance ranking and multivariable Cox significance, brain, liver, lung, and bone metastases were incorporated into the nomogram (**Supplementary Figure 2**). The 3-year CSS calibration curve showed generally acceptable agreement between predicted and observed survival probabilities. These findings suggest that the nomogram provided reasonable calibration for estimating 3-year CSS (**Supplementary Figure 3**).

Using the nomogram score, patients were initially grouped into low-risk (< 3 points), intermediate-risk (3–9 points), and high-risk (≥ 10 points) categories. Metastatic patterns with similar survival were then consolidated into 3 clinically interpretable subgroups: IVB1, metastases excluding brain, liver, lung, and bone involvement; IVB2, metastases involving one of the liver, lung or bone sites, or pairwise combinations of liver-bone or lung-bone sites; and IVB3, brain metastasis or concurrent liver-and-lung metastases. Comparisons among the original metastatic pattern groups showed no significant survival differences within IVB1 or within IVB2 (**Table 2**).

**Table 2:** Description of different metastatic cervical cancer groups based on risk stratification in the training cohort.

Brain involvement	Liver involvement	Lung involvement	Bone involvement	Group	Point	HR (95% CI)	P value	Stage IVB	CSS, median (95% CI), months
No	Yes	Yes	Any	A	≥12	B vs. A	0.333	IVB3	6.0 (5.0, 9.0)
Yes	Any	Any	Any	B	≥10	1.26 (0.79, 2.00)			
No	Yes	No	Yes	C	9	D vs. C 0.99 (0.57, 1.73)	0.977	IVB2	11.0 (10.0, 12.0)
No	No	Yes	Yes	D	9	E vs. D 0.91 (0.56, 1.49)	0.708		
No	Yes	No	No	E	6	F vs. E 0.98 (0.73, 1.33)	0.921		
No	No	Yes	No	F	6	G vs. F 0.89 (0.64, 1.25)	0.511		
No	No	No	Yes	G	3				
No	No	No	No	H	0			IVB1	21.0 (18.0, 25.0)

HR, Hazard Ratio; CI, Confidence Interval; CSS, Cancer-specific survival; Group A, Involvement of liver and lung except brain, regardless of other sites; Group B, Involvement of brain, regardless of other sites; Group C, Involvement of liver and bone except brain or lung, regardless of distant lymph node or other sites; Group D, Involvement of lung and bone except brain or liver, regardless of distant lymph node or other sites; Group E, Liver involvement only, regardless of distant lymph node or other sites; Group F, Lung involvement only, regardless of distant lymph node or other sites; Group G, Bone involvement only, regardless of distant lymph node or other sites; Group H, Involvement of distant lymph node and (or) other sites.

**Figure 2:**
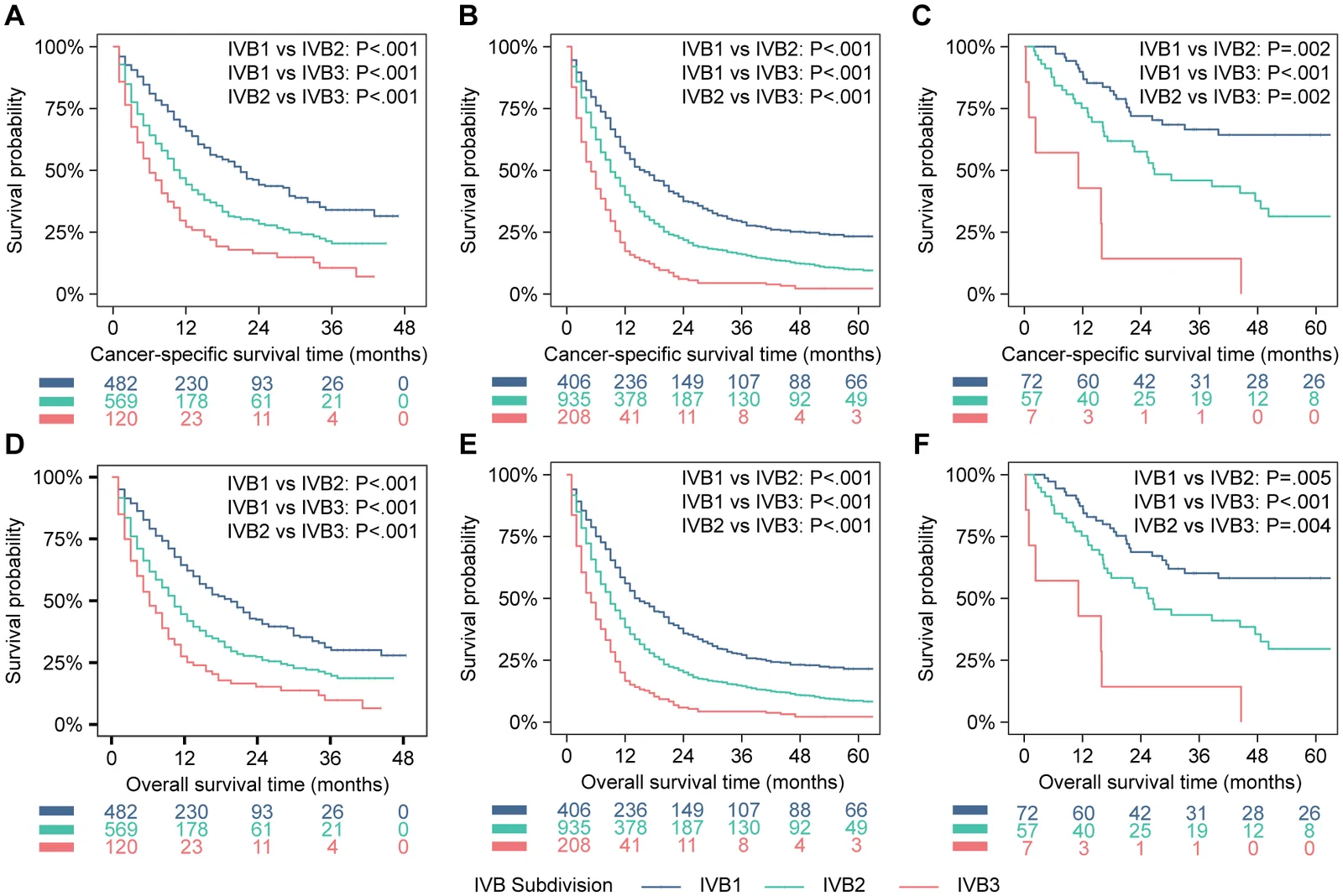
Cancer-specific survival curves for IVB subdivisions in the training cohort **(A)**, internal validation cohort **(B)**, and external validation cohort **(C)**. Overall survival curve for IVB subdivisions in the training cohort **(D)**, the internal validation cohort **(E)**, and the external validation cohort **(F)**. P values were estimated based on the log-rank test.

In the training cohort, median cancer-specific survival decreased from 21 months in IVB1 to 11 months in IVB2 and 6 months in IVB3; the corresponding 3-year cancer-specific survival rates were 34.0%, 20.4%, and 10.6%. Because both the global Schoenfeld residual test and the IVB substage-specific test were significant, indicating non-proportionality of the primary staging variable, a piecewise Cox sensitivity analysis was performed using a 6-month cutoff. After adjustment for race, age, histology, pathological grade, T stage, N stage, and treatment, the prognostic effect of IVB substage was strongest during the first 6 months (IVB2 vs IVB1: HR 1.93, 95% CI 1.49 to 2.50, P < 0.001; IVB3 vs IVB1: HR 2.94, 95% CI 2.06 to 4.20, P < 0.001) and attenuated thereafter, although the associations remained statistically significant after 6 months. Similar gradients were observed for overall survival (**Table 3**; **Figure 2**). In subgroup analyses stratified by age, race, T stage, and histological type, the differences in median cancer-specific survival and median overall survival among the IVB substaging groups were all statistically significant (all P < 0.05) (**Supplementary Table 2**).

**Table 3:** Univariate and multivariate analysis of cancer-specific survival and overall survival.

Subgroups	Train cohort^a^		Internal validation cohort^a^		External validation cohort^b^	
	HR (95% CI)	P value	HR (95% CI)	P value	HR (95% CI)	P value
Univariate						
Part I: CSS						
IVB1	—		—		—	
IVB2	1.75 (1.47, 2.08)	<0.001	1.58 (1.39, 1.81)	<0.001	2.33 (1.36, 4.00)	0.002
IVB3	2.71 (2.11, 3.48)	<0.001	2.87 (2.39, 3.44)	<0.001	8.11 (2.21, 19.27)	<0.001
Part II: OS						
IVB1	—		—		—	
IVB2	1.67 (1.42, 1.97)	<0.001	1.59 (1.40, 1.81)	<0.001	2.01 (1.22, 3.33)	0.006
IVB3	2.53 (1.99, 3.22)	<0.001	2.79 (2.33, 3.33)	<0.001	6.55 (2.83, 15.19)	<0.001
Multivariate						
Part I: CSS						
IVB1	— (0–6 months)		—		—	
IVB2	1.93 (1.49, 2.50)	<0.001	1.32 (1.15, 1.52)	<0.001	1.92 (1.02, 3.59)	0.042
IVB3	2.94 (2.06, 4.20)	<0.001	2.32 (1.92, 2.80)	<0.001	6.52 (2.21, 19.27)	<0.001
IVB1	— (>6 months)					
IVB2	1.35 (1.05, 1.75)	0.021				
IVB3	1.87 (1.17, 2.99)	0.008				
Part II: OS						
IVB1	— (0–6 months)		—		—	
IVB2	1.85 (1.45, 2.36)	<0.001	1.32 (1.15, 1.51)	<0.001	1.58 (0.88, 2.82)	0.124
IVB3	2.68 (1.90, 3.79)	<0.001	2.24 (1.86, 2.69)	<0.001	4.63 (1.64, 13.05)	0.004
IVB1	— (>6 months)					
IVB2	1.26 (0.98, 1.61)	0.071				
IVB3	1.74 (1.10, 2.77)	0.019				

HR, Hazard Ratio; CI, Confidence Interval; CSS, Cancer-specific survival; OS, Overall survival.

^a^The multivariate analysis model was adjusted for age, race, histology, grade, T stage, N stage, and treatment.

^b^The multivariate analysis model was adjusted for age, histology, grade, T stage, N stage, and treatment.

The internal validation cohort showed the same prognostic ordering. Three-year cancer-specific survival rates were 29.0% for IVB1, 16.0% for IVB2, and 4.4% for IVB3, while the corresponding 3-year overall survival rates were 27.1%, 14.6%, and 4.3%. Although the global Schoenfeld residual test was significant, IVB substage did not show evidence of non-proportionality; therefore, conventional Cox estimates for IVB substage were retained. After multivariable adjustment, the IVB subdivision remained associated with both cancer-specific survival (IVB2 vs. IVB1, HR 1.32, P < 0.001; IVB3 vs. IVB1, HR 2.32, P < 0.001) and overall survival (IVB2 vs. IVB1, HR 1.32, P < 0.001; IVB3 vs. IVB1, HR 2.24, P < 0.001) (**Table 3**; **Figure 2**).

Because patients with isolated distant lymph node metastasis were excluded from the internal validation cohort, we assessed their survival in the training cohort to evaluate the potential impact of this exclusion on IVB1 prognostic stratification. Kaplan-Meier analysis showed no significant difference in cancer-specific survival among patients with isolated distant lymph node metastasis, isolated other-site metastasis, or combined distant lymph node and other-site metastases (**Supplementary Figure 4**; log-rank P = 0.460), suggesting broadly comparable outcomes across these non-core-organ metastatic patterns.

In the external validation cohort, the three exploratory IVB subgroups showed a similar directional ordering of survival, with 3-year cancer-specific survival rates of 66.5% in IVB1, 46.0% in IVB2, and 14.3% in IVB3, and corresponding 3-year overall survival rates of 60.2%, 43.3%, and 14.3%, respectively. Neither the global Schoenfeld residual test nor the IVB substage-specific test indicated significant violation of the proportional hazards assumption. In adjusted analyses, the proposed IVB subdivision was associated with cancer-specific survival (IVB2 vs. IVB1, HR 1.92, P = 0.042; IVB3 vs. IVB1, HR 6.52, P < 0.001). IVB3 was also associated with poorer overall survival (HR 4.63, P = 0.004). However, these results should be interpreted cautiously given the small number of patients in the IVB3 subgroup (N = 7) and the lack of patients with brain metastasis (**Table 3**; **Figure 2**).

**Figure 3:**
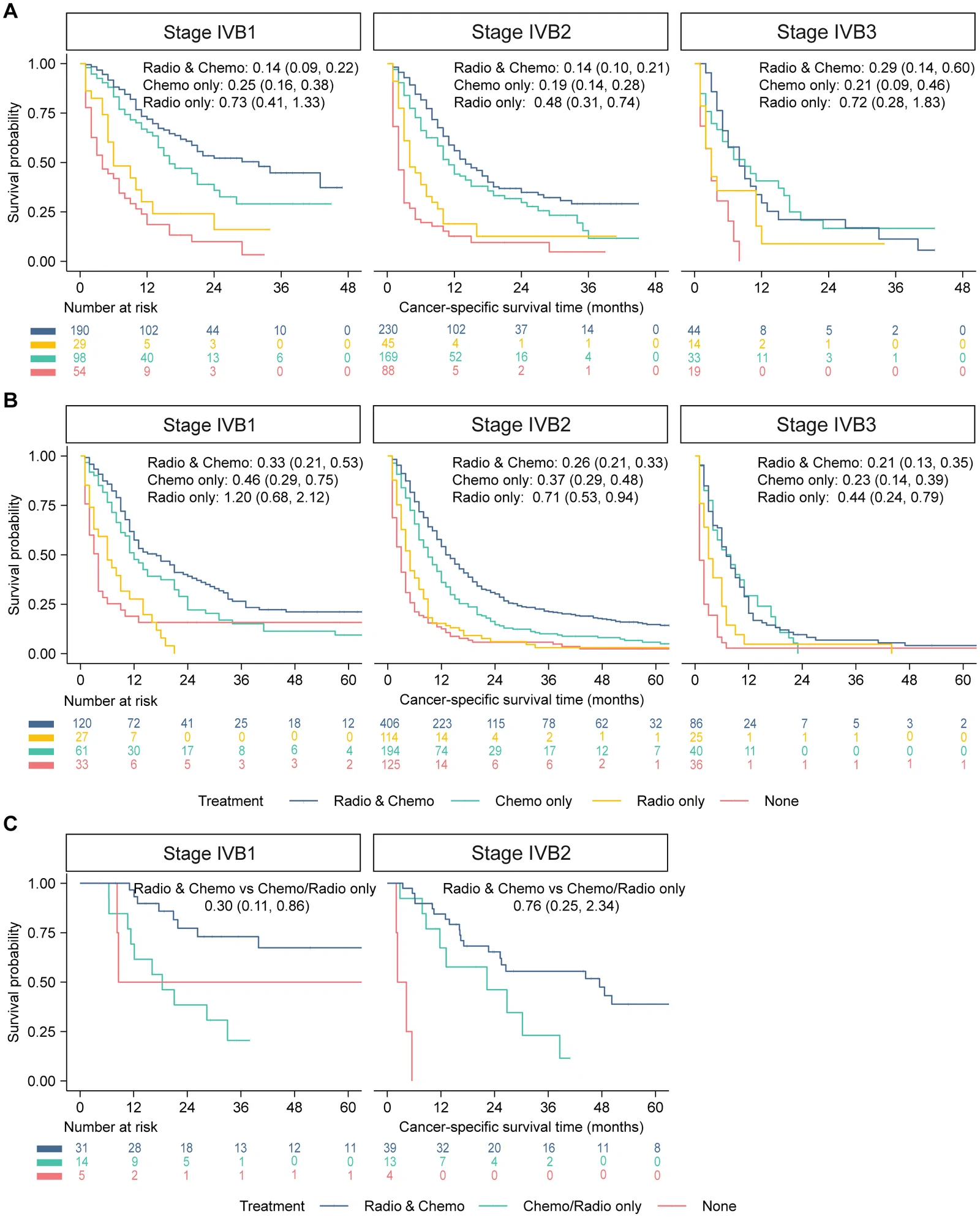
Cancer-specific survival curves stratified by treatment modality in patients with stage IVB1, IVB2, and IVB3 in the training cohort **(A)** and the internal validation cohort **(B)**. Patients who received no treatment were set as the reference category, and all effect measures are reported as HR (95% CI). Cancer-specific survival curves stratified by treatment modality in patients with stage IVB1 and IVB2 in the external validation cohort **(C)**. P values were calculated using the log-rank test. Radio, Radiotherapy; Chemo, Chemotherapy.

Treatment distribution is shown in **Supplementary Table 3**. Among patients who did not undergo surgery, chemotherapy and chemoradiotherapy were associated with longer survival than no treatment in the lower-risk groups. The 3-month landmark Kaplan-Meier analysis according to treatment modality showed survival patterns broadly consistent with the primary analysis (**Supplementary Figure 5**). Schoenfeld residual testing showed no evidence of non-proportionality for the treatment strategy variable in all subgroup Cox models. Global Schoenfeld residual tests were also non-significant in most models, except for the IVB2 subgroup in the internal validation cohort. In the training and internal validation cohorts, chemoradiotherapy was associated with lower observed hazards of death than chemotherapy alone in IVB1 and IVB2 (chemoradiotherapy vs. chemotherapy: training cohort IVB1: HR 0.57, P = 0.007, IVB2: HR 0.74, P = 0.032; internal validation cohort IVB1: HR 0.71, P = 0.080, IVB2: HR 0.70, P < 0.001), whereas no significant survival difference between these approaches was observed in IVB3 (chemoradiotherapy vs. chemotherapy) (**Figure 3**; **Table 4**; **Supplementary Table 4**). Notably, radiotherapy as a standalone treatment exhibited minimal or no association with survival across the subgroups. A similar trend was observed in the external validation cohort, albeit with a limited sample size. These findings were consistent across the entire population (**Supplementary Table 5**).

**Table 4:** Multivariate analysis of association of cancer-specific survival with radiotherapy and chemotherapy in patients who did not receive surgery.

Training cohort	Model 1		Model 2		Model 3	
	HR (95% CI)	P value	HR (95% CI)	P value	HR (95% CI)	P value
Train Cohort^a^						
IVB1						
None	Reference					
Radiotherapy only	0.73 (0.41, 1.33)	0.308	Reference			
Chemotherapy only	0.25 (0.16, 0.38)	<0.001	0.33 (0.18, 0.61)	<0.001	Reference	
Radio & Chemo	0.14 (0.09, 0.22)	<0.001	0.19 (0.11, 0.34)	<0.001	0.57 (0.38, 0.86)	0.007
IVB2						
None	Reference					
Radiotherapy only	0.48 (0.31, 0.74)	0.001	Reference			
Chemotherapy only	0.19 (0.14, 0.28)	<0.001	0.41 (0.27, 0.62)	<0.001	Reference	
Radio & Chemo	0.14 (0.10, 0.21)	<0.001	0.30 (0.20, 0.46)	<0.001	0.74 (0.57, 0.98)	0.032
IVB3						
None	Reference					
Radiotherapy only	0.72 (0.28, 1.83)	0.489	Reference			
Chemotherapy only	0.21 (0.09, 0.46)	<0.001	0.29 (0.13, 0.66)	0.003	Reference	
Radio & Chemo	0.29 (0.14, 0.60)	<0.001	0.40 (0.18, 0.89)	0.025	1.37 (0.75, 2.50)	0.308

HR, Hazard Ratio; CI, Confidence Interval; Radio, Radiotherapy; Chemo, Chemotherapy.

^a^The multivariate analysis model was adjusted for age, race, histology, grade, T stage and N stage.

Additional analyses were conducted in the external validation cohort to evaluate the association of metastatic-site radiotherapy and immunotherapy with survival. Among patients who received radiotherapy to the primary site, 55 also underwent radiotherapy to metastatic sites, whereas 40 received radiotherapy to the primary site alone. KM analyses showed no significant survival difference between patients treated with and without metastatic-site radiotherapy in the overall cohort, and similar results were observed in the stage IVB1 and IVB2 subgroups (**Supplementary Figure 6**). Furthermore, 33 patients received immunotherapy, and 103 did not. No significant survival difference associated with immunotherapy was observed in the overall cohort or in the stage IVB1 and IVB2 subgroups (**Supplementary Figure 7**).

Further analyses explored the association of surgery with survival in patients with stage IVB cervical cancer. Compared with no primary-site surgery, primary-site surgery was associated with longer survival among patients with stage IVB1 and, in selected analyses, stage IVB2 disease across the training and validation cohorts (**Supplementary Figure 8**). Notably, in the 3-month landmark Kaplan-Meier analyses, significant associations were observed only in the validation cohorts (**Supplementary Figure 9**). No clear survival association for primary site surgery was observed in stage IVB3 in either the training or internal validation cohorts (**Supplementary Table 6**, **Supplementary Figures 8, 9**). Metastatic site surgery was associated with longer survival only in stage IVB1 in the training and external validation cohorts, but this finding was not consistently reproduced across datasets. In contrast, no evident survival association was observed for metastatic site surgery in stage IVB2 or IVB3 disease (**Supplementary Figure 10**, **Supplementary Table 6**). The 3-month landmark Kaplan-Meier analyses for distant metastatic site surgery showed survival patterns generally consistent with the primary analyses (**Supplementary Figure 11**).

## Discussion

Using a large cohort of *de novo* stage IVB cervical cancer, we identified brain, liver, lung, and bone metastases as principal survival determinants via random survival forest and Cox regression, and developed a three-tier prognostic framework (IVB1–IVB3) based on their presence and combinations. The three tiers showed distinct cancer-specific survival in training and internal validation. External validation supported IVB1/IVB2 separation but was underpowered for IVB3 and brain metastasis due to limited IVB3 cases. Overall, these findings suggest that metastatic patterns reflect clinically relevant prognostic heterogeneity in stage IVB cervical cancer, highlighting the need for prospective multicenter validation.

The proposed subdivision reflects the substantial heterogeneity within stage IVB cervical cancer. Prior reports have shown that brain, liver, and lung metastases are associated with poor outcomes in newly diagnosed stage IVB cervical cancer, with brain metastasis conferring particularly unfavorable survival (3, 15). Our findings showed that synchronous liver and lung metastases intensified the prognostic burden, markedly reducing survival similarly to brain metastasis cases, which aligns with other evidence (16). Consequently, these groups were categorized as the poorest-prognosis stage IVB3. In our analysis, an elevated mortality risk was observed specifically with synchronous liver and lung metastases, but not with bone metastases combined with liver or lung involvement. Previous studies have also indicated better survival for lymph node-only or unknown-site metastases compared to single or multiple organ metastases (12, 17). In line with these observations, patients without brain, liver, lung, or bone metastases were classified as the lowest-risk IVB1 subgroup. Taken together, these findings suggest that metastatic sites and metastatic combinations may provide additional prognostic information within stage IVB cervical cancer and support further evaluation of this approach as an investigational prognostic framework.

Brain, liver, lung, and bone metastases are among the most commonly evaluated distant metastatic sites in cervical cancer (15). In our study, both Cox regression and random survival forest analyses further identified these metastatic sites as major contributors to cancer-specific survival, supporting their inclusion in the proposed IVB subdivision. Their distinct prognostic effects may reflect differences in metastatic tropism, organ-specific microenvironments, and the biological characteristics of metastatic tumor clones (18, 19). The particularly poor prognosis associated with brain metastases may reflect aggressive tumor biology, limited penetration of systemic therapies across the blood–brain barrier, and the potentially severe consequences of intracranial progression (20, 21). Further studies are warranted to elucidate the biological mechanisms underlying metastatic-site preferences and their prognostic heterogeneity.

Recent studies have developed prognostic models for patients with stage IVB cervical cancer. Using SEER data, Zhang et al. developed a nomogram for overall survival in newly diagnosed metastatic cervical cancer. Although the model stratified patients into three risk groups, it primarily incorporated the number of metastatic sites and did not fully account for site-specific prognostic differences (11). In addition, distant lymph node and other-site metastases were not incorporated, which may limit its applicability to the full spectrum of stage IVB disease. Another model classified metastatic patterns into lymph node-only, organ-only, organ plus lymph node, or unknown-site metastases, but similarly did not distinguish prognosis according to specific metastatic organs (12). In contrast, our proposed subdivision uses routinely available metastatic-site data and emphasizes the distinct prognostic impact of brain, liver, lung, and bone involvement. Compared with more complex nomograms, this three-tier framework may be relatively straightforward to evaluate in future prospective studies.

Patients with stage IVB cervical cancer have traditionally been managed primarily with systemic therapy. However, increasing evidence suggests that selected patients may benefit from locoregional treatment, such as radiotherapy or surgery (22, 23). Nevertheless, optimal patient selection remains unclear. Our metastatic pattern-based stratification provides an exploratory framework for generating hypotheses regarding the potential role of locoregional treatment across subgroups.

Radiotherapy has an established role in symptom palliation and local disease control in advanced cervical cancer (24, 25). Several retrospective studies have suggested that radiotherapy may be associated with improved survival in selected patients with stage IVB cervical cancer (24, 25). Moreover, Perkins et al. found no increased complications with the addition of pelvic radiation (26). In our observational analysis, chemoradiotherapy was associated with longer survival than chemotherapy alone in IVB1 and IVB2, whereas no statistically significant association between these treatment approaches was observed in IVB3. The SEER database does not contain several important clinical determinants of treatment selection, including ECOG performance status and detailed metastatic burden. Consequently, residual confounding and treatment-selection bias cannot be excluded and may have contributed to the observed associations. Therefore, these findings should be interpreted as exploratory associations rather than evidence of a treatment effect.

Surgery is not routinely recommended for unselected patients with advanced cervical cancer, although primary tumor resection may be considered in carefully selected metastatic cases. Previous retrospective studies have suggested that local surgery may be associated with improved survival in stage IVB cervical cancer (27). In our observational analysis, primary-site surgery was associated with longer survival in IVB1 and in selected analyses of IVB2 disease, whereas no clear survival association was observed in IVB3 disease. These findings are clinically plausible, as patients with less extensive metastatic involvement may be more likely to benefit from local disease control. However, these associations may be subject to residual confounding and treatment-selection bias and should therefore not be interpreted as evidence that primary-site surgery confers a survival benefit in these subgroups.

Metastasectomy is another local treatment approach that may be considered in highly selected patients with stage IVB cervical cancer. Ki et al. reported longer median overall survival in patients with ≤3 metastatic lesions than in those with >4 lesions (19). In our analysis, metastatic-site surgery was associated with longer survival only in IVB1 in the training cohort, and this finding was not reproduced in the internal validation cohort. Hence, prospective studies are required to clarify the association of metastatic-site surgery with survival in IVB1 patients. No clear survival association was observed in IVB2 or IVB3. This may reflect differences in metastatic burden and disease biology, as patients with IVB1 disease may have less aggressive metastatic patterns, whereas vital organ involvement in IVB2 and IVB3 may reduce the impact of metastasectomy. Consistently, another study reported no significant survival difference between patients treated with and without other-site surgery (28).

Platinum-based chemotherapy has been shown to improve outcomes in recurrent or metastatic disease (29). In the present study, chemotherapy-containing treatment was associated with improved survival in several subgroups, whereas radiotherapy alone showed limited or no survival association. These findings reinforce the central role of systemic therapy in stage IVB disease.

Our IVB1-IVB3 classification complements the emerging oligometastatic paradigm by capturing the prognostic heterogeneity of metastatic organ patterns. However, IVB1/IVB2 should not be equated with oligometastatic disease because metastatic burden and the feasibility of definitive local treatment were unavailable in SEER. In the era of immunotherapy, the benefit of immunotherapy may vary across clinical subgroups, with less clear benefit reported in patients with metastatic disease at initial diagnosis (30, 31). Similarly, atezolizumab did not show statistically significant survival improvement in patients with stage IVB cervical cancer (32). Consistently, immunotherapy use was not significantly associated with survival in our external cohort. Further prospective studies are needed to determine whether IVB1-IVB3 subgroups differ in their response to immunotherapy.

Notably, the external cohort showed substantially higher overall survival than both SEER cohorts. This marked difference suggests that the external cohort was not directly comparable with the population-based SEER cohorts and may reflect differences in single-center referral patterns, baseline health status, treatment intensity, and regional treatment practices. Such differences may introduce selection and referral bias and limit the generalizability and international transportability of the proposed framework. In addition, the IVB3 subgroup in the external cohort comprised only 7 patients, and no patient with brain metastasis was represented, despite brain metastasis receiving the highest individual prognostic weight in the nomogram. As a result, our external assessment of the IVB3 category, and particularly of brain metastasis, is statistically underpowered and should be viewed as preliminary. Definitive confirmation of the prognostic hierarchy will require larger, more diverse cohorts.

This study has several limitations. First, the SEER database lacks key factors that influence treatment selection, including performance status, comorbidities, and metastatic burden, resulting in potential treatment-selection bias and residual confounding. These factors may have led to an overestimation of the apparent survival benefit of local treatment, particularly in the IVB1 and IVB2 subgroups. Thus, treatment-related findings should be interpreted as observational associations rather than evidence of treatment benefit. Second, although the framework was externally evaluated, the external cohort was relatively small and differed from the SEER cohorts in survival, treatment patterns, and clinical setting, which may limit generalizability and explain the inconsistent association between metastatic-site surgery and cancer-specific survival in IVB1 patients. Third, SEER does not provide detailed anatomical information for metastases classified as other. As a result, IVB1, which includes patients with non-core-organ distant metastases, represents a heterogeneous category rather than a biologically uniform subgroup. Moreover, metastatic burden may influence prognosis beyond metastatic location, but detailed burden data are not comprehensively available in SEER. Larger prospective cohorts with granular clinical, treatment, and molecular data are needed to validate the proposed subdivision and treatment-related findings.

## Conclusion

This study developed an investigational prognostic framework that uses metastatic patterns to characterize prognostic heterogeneity within stage IVB cervical cancer. The IVB1-IVB3 framework demonstrated prognostic ordering in the population-based training and internal validation cohorts, while findings in the independent single-center cohort remain exploratory because of substantial differences in cohort characteristics, limited IVB3 sample size, and absence of brain metastases. Treatment-related analyses should be interpreted as observational, hypothesis-generating associations rather than evidence supporting a specific treatment strategy. Prospective multicenter validation in geographically and clinically diverse populations, with adequate representation of high-risk metastatic patterns and comprehensive clinical and treatment-selection variables, is required before this framework can be considered for clinical adoption.

## Data Availability

The original contributions presented in the study are included in the article/**Supplementary Material**. Further inquiries can be directed to the corresponding authors.
