# Supplementary material for: An investigational prognostic framework for stage IVB cervical cancer based on metastatic patterns: a population-based study with exploratory external evaluation
Source: Front Oncol. 2026 Sep 3;16:1931638. doi: 10.3389/fonc.2026.1931638 (PMC13581830; doi:10.3389/fonc.2026.1931638)
Supplement: Supplementary file 1 [file SupplementaryFile1.docx]

**Table S1 Univariable and multivariable cox regression analysis of cancer-specific survival in the training cohort.**

| **Characteristic** | **Univariable** | | **Multivariable** | |
| --- | --- | --- | --- | --- |
|  | HR (95% CI) | **P value** | HR (95% CI) | **P value** |
| **Race** |  |  |  |  |
| Black | Reference |  | Reference |  |
| Other | 0.68 (0.50, 0.93) | 0.014 | 0.74 (0.54, 1.01) | 0.055 |
| White | 0.95 (0.78, 1.16) | 0.636 | 1.05 (0.86, 1.29) | 0.644 |
| **Age, years** |  |  |  |  |
| ˂ 60 | Reference |  | Reference |  |
| >= 60 | 1.35 (1.16, 1.58) | <0.001 | 1.28 (1.10, 1.50) | 0.002^*^ |
| **Histology** |  |  |  |  |
| SCC | Reference |  | — | — |
| Non-SCC | 1.19 (1.02, 1.39) | 0.030 | — | — |
| **Histologic grade** |  |  |  |  |
| Grade I | Reference |  | Reference |  |
| Grade II | 1.05 (0.56, 1.95) | 0.887 | 1.15 (0.62, 2.16) | 0.655 |
| Grade III-IV | 1.59 (0.87, 2.91) | 0.131 | 1.60 (0.87, 2.94) | 0.130 |
| Unknown | 1.51 (0.82, 2.75) | 0.183 | 1.54 (0.84, 2.82) | 0.165 |
| **T stage** |  |  |  |  |
| T1-2 | Reference |  | Reference |  |
| T3-4 | 1.32 (1.09, 1.58) | 0.004 | 1.19 (0.98, 1.43) | 0.074 |
| Tx | 1.56 (1.24, 1.95) | <0.001 | 1.32 (1.04, 1.67) | 0.020^*^ |
| **N stage** |  |  |  |  |
| N0 | Reference |  | — | — |
| N1-2 | 1.00 (0.83, 1.21) | 0.99 | — | — |
| Nx | 1.21 (0.91, 1.60) | 0.181 | — | — |
| **Distant lymph node metastasis** | |  |  |  |
| No | Reference |  | Reference |  |
| Yes | 1.03 (0.88, 1.20) | 0.731 | 1.15 (0.98, 1.35) | 0.088 |
| **Distant other sites metastasis** | |  |  |  |
| No | Reference |  | Reference |  |
| Yes | 1.07 (0.91, 1.26) | 0.391 | 1.17 (0.99, 1.39) | 0.058^*^ |
| **Bone metastasis** |  |  |  |  |
| No | Reference |  | Reference |  |
| Yes | 1.36 (1.14, 1.62) | <0.001 | 1.28 (1.07, 1.53) | 0.008^*^ |
| **Brain metastasis** |  |  |  |  |
| No | Reference |  | Reference |  |
| Yes | 2.24 (1.50, 3.34) | <0.001 | 1.74 (1.16, 2.63) | 0.008^*^ |
| **Liver metastasis** |  |  |  |  |
| No | Reference |  | Reference |  |
| Yes | 1.57 (1.31, 1.89) | <0.001 | 1.42 (1.17, 1.72) | <0.001^*^ |
| **Lung metastasis** |  |  |  |  |
| No | Reference |  | Reference |  |
| Yes | 1.64 (1.40, 1.92) | <0.001 | 1.52 (1.29, 1.80) | <0.001 |

Abbreviations: HR, Hazard Ratio; CI, Confidence Interval.

Significance: * P value < 0.05.

**Table S2 Stratified analysis by T stage, age, histology, and race based on the IVB subcategory in the training cohort.**

| Characteristic | IVB Subcategory |  | CSS | | OS | |
| --- | --- | --- | --- | --- | --- | --- |
|  |  | N (%) | Median (95% CI),  months | P value | Median (95% CI),  months | P value |
| **T stage** |  |  |  |  |  |  |
| T1-2 | IVB1 | 167 (14.3) | 29.0 (21.0, NA) | <0.001 | 29.0 (20.0, NA) | <0.001 |
|  | IVB2 | 164 (14.0) | 11.0 (9.0, 14.0) |  | 11.0 (9.0, 13.0) |  |
|  | IVB3 | 31 (2.6) | 6.0 (5.0, NA) |  | 6.0 (5.0, NA) |  |
| T3-4 | IVB1 | 244 (20.8) | 19.0 (14.0, 24.0) | <0.001 | 15.0 (13.0, 21.0) | <0.001 |
|  | IVB2 | 297 (25.4) | 11.0 (10.0, 14.0) |  | 10.0 (9.0, 12.0) |  |
|  | IVB3 | 52 (4.4) | 5.0 (3.0, 9.0) |  | 5.0 (3.0, 8.0) |  |
| Tx | IVB1 | 71 (6.1) | 13.0 (10.0, NA) | 0.022 | 12.0 (10.0, NA) | 0.012 |
|  | IVB2 | 108 (9.2) | 9.0 (5.0, 13.0) |  | 7.0 (5.0, 10.0) |  |
|  | IVB3 | 37 (3.2) | 8.0 (5.0, 16.0) |  | 8.0 (5.0, 15.0) |  |
| **Age** |  |  |  |  |  |  |
| <60 | IVB1 | 295 (25.2) | 22.0 (18.0, 29.0) | <0.001 | 21.0 (16.0, 25.0) | <0.001 |
|  | IVB2 | 323 (27.6) | 13.0 (11.0, 17.0) |  | 12.0 (10.0, 15.0) |  |
|  | IVB3 | 69 (5.9) | 6.0 (5.0, 11.0) |  | 6.0 (5.0, 9.0) |  |
| >=60 | IVB1 | 187 (16) | 20.0 (13.0, 35.0) | <0.001 | 13.0 (11.0, 21.0) | <0.001 |
|  | IVB2 | 246 (21) | 8.0 (6.0, 10.0) |  | 8.0 (6.0, 10.0) |  |
|  | IVB3 | 51 (4.4) | 8.0 (4.0, 11.0) |  | 8.0 (4.0, 10.0) |  |
| **Histology** |  |  |  |  |  |  |
| SCC | IVB1 | 306 (26.1) | 21.0 (16.0, 30.0) | <0.001 | 19.0 (15.0, 24.0) | <0.001 |
|  | IVB2 | 342 (29.2) | 11.0 (10.0, 13.0) |  | 10.0 (9.0, 12.0) |  |
|  | IVB3 | 68 (5.8) | 8.0 (5.0, 11.0) |  | 8.0 (5.0, 11.0) |  |
| Non-SCC | IVB1 | 176 (15) | 21.0 (17.0, 29.0) | <0.001 | 19.0 (14.0, 25.0) | <0.001 |
|  | IVB2 | 227 (19.4) | 10.0 (8.0, 13.0) |  | 9.0 (7.0, 12.0) |  |
|  | IVB3 | 52 (4.4) | 5.0 (3.0, 8.0) |  | 5.0 (3.0, 8.0) |  |
| **Race** |  |  |  |  |  |  |
| Black | IVB1 | 75 (6.4) | 23.0 (16.0, NA) | <0.001 | 20.0 (15.0, 29.0) | 0.001 |
|  | IVB2 | 104 (8.9) | 9.0 (7.0, 12.0) |  | 7.0 (6.0, 11.0) |  |
|  | IVB3 | 33 (2.8) | 8.0 (4.0, NA) |  | 8.0 (4.0, 40.0) |  |
| White | IVB1 | 352 (30.1) | 20.0 (16.0, 22.0) | <0.001 | 18.0 (14.0, 22.0) | <0.001 |
|  | IVB2 | 403 (34.4) | 10.0 (9.0, 12.0) |  | 10.0 (9.0, 11.0) |  |
|  | IVB3 | 73 (6.2) | 6.0 (4.0, 9.0) |  | 6.0 (4.0, 8.0) |  |
| Other | IVB1 | 55 (4.7) | 25.0 (24.0, NA) | 0.001 | 24.0 (14.0, NA) | 0.009 |
|  | IVB2 | 62 (5.3) | 18.0 (13.0, 35.0) |  | 18.0 (12.0, 35.0) |  |
|  | IVB3 | 14 (1.2) | 9.0 (5.0, NA) |  | 9.0 (5.0, NA) |  |

Abbreviations: N, number; CI, Confidence Interval.

**Table S3 Baseline patient treatment characteristics by IVB subcategory.**

|  | **Training Cohort** | | | **Internal Validation Cohort** | | | **External Validation Cohort** | | |
| --- | --- | --- | --- | --- | --- | --- | --- | --- | --- |
|  | **IVB1 N (%)** | **IVB2 N (%)** | **IVB3 N (%)** | **IVB1 N (%)** | **IVB2 N (%)** | **IVB3 N (%)** | **IVB1 N (%)** | **IVB2 N (%)** | **IVB3 N (%)** |
| **Total** | 482 | 569 | 120 | 406 | 935 | 208 | 72 | 57 | 7 |
| **Surgery to primary site** | |  |  |  |  |  |  |  |  |
| Yes | 75 (15.6) | 15 (2.64) | 3 (2.50) | 119 (29.3) | 49 (5.24) | 7 (3.37) | 22 (30.6) | 1 (1.75) | 0 (0.00) |
| No | 407 (84.4) | 554 (97.4) | 117 (97.5) | 287 (70.7) | 886 (94.8) | 201 (96.6) | 50 (69.4) | 56 (98.2) | 7 (100.0) |
| **Surgery to distant sites** | |  |  |  |  |  |  |  |  |
| Yes | 58 (12.0) | 24 (4.22) | 7 (5.83) | 82 (20.2) | 56 (6.00) | 14 (6.73) | 20 (27.8) | 0 (0.00) | 0 (0.00) |
| No | 424 (88.0) | 545 (95.8) | 113 (94.2) | 324 (79.8) | 879 (94.0) | 194 (93.3) | 52 (72.2) | 57 (100.0) | 7 (100.0) |
| **Radiation** |  |  |  |  |  |  |  |  |  |
| Yes | 283 (58.7) | 289 (50.8) | 63 (52.5) | 234 (57.6) | 571 (61.1) | 122 (58.7) | 50 (69.4) | 43 (75.4) | 2 (28.6) |
| No/Unknown | 199 (41.3) | 280 (49.2) | 57 (47.5) | 172 (42.4) | 364 (38.9) | 86 (41.3) | 22 (30.6) | 14 (24.6) | 5 (71.4) |
| **Chemotherapy** |  |  |  |  |  |  |  |  |  |
| Yes | 388 (80.5) | 431 (75.7) | 85 (70.8) | 312 (76.8) | 678 (72.5) | 143 (68.8) | 67 (93.1) | 50 (87.7) | 5 (71.4) |
| No/Unknown | 94 (19.5) | 138 (24.3) | 35 (29.2) | 94 (23.2) | 257 (27.5) | 65 (31.3) | 5 (6.94) | 7 (12.3) | 2 (28.6) |

Abbreviations: N, number.

**Table S4 Multivariate analysis of association of cancer-specific survival with radiotherapy and chemotherapy in patients who did not receive surgery.**

|  | Model 1 | | Model 2 | | Model 3 | |
| --- | --- | --- | --- | --- | --- | --- |
|  | HR (95% CI) | **P value** | HR (95% CI) | **P value** | HR (95% CI) | **P value** |
| Internal Validation Cohort^a^ | |  |  |  |  |  |
| IVB1 |  |  |  |  |  |  |
| None | Reference |  |  |  |  |  |
| Radiotherapy only | 1.20 (0.68, 2.12) | 0.532 | Reference |  |  |  |
| Chemotherapy only | 0.46 (0.29, 0.75) | 0.002 | 0.39 (0.23, 0.66) | <0.001 | Reference |  |
| Radio & Chemo | 0.33 (0.21, 0.53) | <0.001 | 0.28 (0.17, 0.45) | <0.001 | 0.71 (0.49, 1.04) | 0.080 |
| IVB2 |  |  |  |  |  |  |
| None | Reference |  |  |  |  |  |
| Radiotherapy only | 0.71 (0.53, 0.94) | 0.018 | Reference |  |  |  |
| Chemotherapy only | 0.37 (0.29, 0.48) | <0.001 | 0.52 (0.40, 0.68) | <0.001 | Reference |  |
| Radio & Chemo | 0.26 (0.21, 0.33) | <0.001 | 0.37 (0.29, 0.46) | <0.001 | 0.70 (0.57, 0.85) | <0.001 |
| IVB3 |  |  |  |  |  |  |
| None | Reference |  |  |  |  |  |
| Radiotherapy only | 0.44 (0.24, 0.79) | 0.006 | Reference |  |  |  |
| Chemotherapy only | 0.23 (0.14, 0.39) | <0.001 | 0.53 (0.30, 0.92) | 0.025 | Reference |  |
| Radio & Chemo | 0.21 (0.13, 0.35) | <0.001 | 0.49 (0.30, 0.79) | 0.004 | 0.92 (0.61, 1.39) | 0.700 |
| External Validation Cohort^b^ | |  |  |  |  |  |
| IVB1 |  |  |  |  |  |  |
| None | Reference |  |  |  |  |  |
| Radio/Chemo only | 0.59 (0.10, 3.45) | 0.556 | Reference |  |  |  |
| Radio & Chemo | 0.18 (0.03, 1.05) | 0.056 | 0.30 (0.11, 0.86) | 0.025 |  |  |
| IVB2 |  |  |  |  |  |  |
| None | Reference |  |  |  |  |  |
| Radio/Chemo only | 0.03 (0.00, 0.18) | <0.001 | Reference |  |  |  |
| Radio & Chemo | 0.02 (0.00, 0.14) | <0.001 | 0.76 (0.25, 2.34) | 0.632 |  |  |

Abbreviations: HR, Hazard Ratio; CI, Confidence Interval; Radio, Radiotherapy; Chemo, Chemotherapy.

^a^The multivariate analysis model was adjusted for age, race, histology, grade, T stage and N stage.

^b^The multivariate analysis model was adjusted for age, histology, grade, T stage and N stage.

**Table S5 Multivariate analysis of association of cancer-specific survival with radiotherapy and chemotherapy in all patients.**

|  | Model 1 | | Model 2 | | Model 3 | |
| --- | --- | --- | --- | --- | --- | --- |
|  | HR (95% CI) | **P value** | HR (95% CI) | **P value** | HR (95% CI) | **P value** |
| Train Cohort^a^ |  |  |  |  |  |  |
| IVB1 |  |  |  |  |  |  |
| None | Reference |  |  |  |  |  |
| Radiotherapy only | 0.61 (0.35, 1.06) | 0.077 | Reference |  |  |  |
| Chemotherapy only | 0.22 (0.15, 0.33) | <0.001 | 0.36 (0.21, 0.62) | <0.001 | Reference |  |
| Radio & Chemo | 0.15 (0.10, 0.23) | <0.001 | 0.25 (0.15, 0.43) | <0.001 | 0.69 (0.48, 0.99) | 0.046 |
| IVB2 |  |  |  |  |  |  |
| None | Reference |  |  |  |  |  |
| Radiotherapy only | 0.50 (0.33, 0.77) | 0.001 | Reference |  |  |  |
| Chemotherapy only | 0.19 (0.13, 0.26) | <0.001 | 0.37 (0.25, 0.56) | <0.001 | Reference |  |
| Radio & Chemo | 0.14 (0.10, 0.20) | <0.001 | 0.29 (0.19, 0.44) | <0.001 | 0.78 (0.60, 1.02) | 0.068 |
| IVB3 |  |  |  |  |  |  |
| None | Reference |  |  |  |  |  |
| Radiotherapy only | 0.60 (0.25, 1.44) | 0.252 | Reference |  |  |  |
| Chemotherapy only | 0.23 (0.11, 0.49) | <0.001 | 0.39 (0.18, 0.85) | 0.018 | Reference |  |
| Radio & Chemo | 0.24 (0.12, 0.47) | <0.001 | 0.39 (0.18, 0.84) | 0.017 | 1.01 (0.59, 1.72) | 0.985 |
| Internal Validation Cohort^a^ | |  |  |  |  |  |
| IVB1 |  |  |  |  |  |  |
| None | Reference |  |  |  |  |  |
| Radiotherapy only | 0.93 (0.59, 1.49) | 0.771 | Reference |  |  |  |
| Chemotherapy only | 0.48 (0.33, 0.69) | <0.001 | 0.51 (0.33, 0.79) | 0.002 | Reference |  |
| Radio & Chemo | 0.34 (0.24, 0.48) | <0.001 | 0.37 (0.25, 0.55) | <0.001 | 0.72 (0.54, 0.96) | 0.025 |
| IVB2 |  |  |  |  |  |  |
| None | Reference |  |  |  |  |  |
| Radiotherapy only | 0.80 (0.61, 1.05) | 0.102 | Reference |  |  |  |
| Chemotherapy only | 0.40 (0.32, 0.51) | <0.001 | 0.50 (0.39, 0.65) | <0.001 | Reference |  |
| Radio & Chemo | 0.28 (0.22, 0.35) | <0.001 | 0.35 (0.28, 0.44) | <0.001 | 0.70 (0.58, 0.83) | <0.001 |
| IVB3 |  |  |  |  |  |  |
| None | Reference |  |  |  |  |  |
| Radiotherapy only | 0.55 (0.31, 0.97) | 0.039 | Reference |  |  |  |
| Chemotherapy only | 0.26 (0.16, 0.43) | <0.001 | 0.48 (0.28, 0.81) | 0.006 | Reference |  |
| Radio & Chemo | 0.26 (0.17, 0.41) | <0.001 | 0.47 (0.30, 0.76) | 0.002 | 1.00 (0.68, 1.46) | 0.980 |
| External Validation^b^ Cohort | |  |  |  |  |  |
| IVB1 |  |  |  |  |  |  |
| None | Reference |  |  |  |  |  |
| Radio/Chemo only | 0.52 (0.09, 2.93) | 0.556 | Reference |  |  |  |
| Radio & Chemo | 0.14 (0.02, 0.75) | 0.022 | 0.26 (0.09, 0.74) | 0.012 |  |  |
| IVB2 |  |  |  |  |  |  |
| None | Reference |  |  |  |  |  |
| Radio/Chemo only | 0.01 (0.00, 0.10) | <0.001 | Reference |  |  |  |
| Radio & Chemo | 0.03 (0.00, 0.19) | <0.001 | 0.50 (0.19, 1.31) | 0.157 |  |  |

Abbreviations: HR, Hazard Ratio; CI, Confidence Interval; Radio, Radiotherapy; Chemo, Chemotherapy.

^a^The multivariate analysis model was adjusted for age, race, histology, grade, T stage and N stage.

^b^The multivariate analysis model was adjusted for age, histology, grade, T stage and N stage.

**Table S6 Multivariate analysis of association of cancer-specific survival with treatment modality.**

|  | IVB1 | | IVB2 | | IVB3 | |
| --- | --- | --- | --- | --- | --- | --- |
|  | HR (95% CI) | **P value** | HR (95% CI) | **P value** | HR (95% CI) | **P value** |
| **Train Cohort** |  |  |  |  |  |  |
| **Surgery to primary site** | |  |  |  |  |  |
| No | Reference |  | Reference |  | Reference |  |
| Yes | 0.55 (0.35, 0.86) | 0.008^*^ | 0.52 (0.23, 1.18) | 0.119 | 0.56 (0.12, 2.58) | 0.454 |
| **Surgery to distant sites** | |  |  |  |  |  |
| No | Reference |  | Reference |  | Reference |  |
| Yes | 0.57 (0.34, 0.96) | 0.033^*^ | 0.56 (0.28, 1.11) | 0.096 | 1.52 (0.60, 3.81) | 0.377 |
| **Radiotherapy** |  |  |  |  |  |  |
| No/Unknown | Reference |  | Reference |  | Reference |  |
| Yes | 0.64 (0.48, 0.87) | 0.004^*^ | 0.65 (0.52, 0.82) | <0.001^*^ | 0.86 (0.74, 1.85) | 0.506 |
| **Chemotherapy** |  |  |  |  |  |  |
| No/Unknown | Reference |  | Reference |  | Reference |  |
| Yes | 0.24 (0.17, 0.34) | <0.001^*^ | 0.22 (0.17, 0.30) | <0.001^*^ | 0.29 (0.17, 0.51) | <0.001^*^ |
| **Internal Validation Cohort** | |  |  |  |  |  |
| **Surgery to primary site** | |  |  |  |  |  |
| No | Reference |  | Reference |  | Reference |  |
| Yes | 0.44 (0.32, 0.60) | <0.001^*^ | 0.53 (0.37, 0.75) | <0.001^*^ | 0.83 (0.38, 1.81) | 0.638 |
| **Surgery to distant sites** | |  |  |  |  |  |
| No | Reference |  | Reference |  | Reference |  |
| Yes | 0.99 (0.73, 1.33) | 0.947 | 0.89 (0.67, 1.20) | 0.446 | 1.06 (0.57, 1.97) | 0.866 |
| **Radiotherapy** |  |  |  |  |  |  |
| No/Unknown | Reference |  | Reference |  | Reference |  |
| Yes | 0.70 (0.54, 0.91) | 0.007^*^ | 0.70 (0.60, 0.82) | <0.001^*^ | 0.83 (0.60, 1.15) | 0.255 |
| **Chemotherapy** |  |  |  |  |  |  |
| No/Unknown | Reference |  | Reference |  | Reference |  |
| Yes | 0.42 (0.32, 0.55) | <0.001^*^ | 0.38 (0.32, 0.44) | <0.001^*^ | 0.36 (0.26, 0.51) | <0.001^*^ |

Abbreviations: HR, Hazard Ratio; CI, Confidence Interval.

Significance: * P value < 0.05.

Figure S1. Variable Importance in the Random Survival Forest Model.

Figure S2. Nomogram for predicting cancer-specific survival and risk stratification in patients with de novo metastatic cervical cancer based on metastatic sites.

Figure S3. Calibration curve of the nomogram for predicting 3-year cancer-specific survival. The x-axis represents the nomogram-predicted probability of 3-year cancer-specific survival, and the y-axis represents the observed 3-year cancer-specific survival estimated using the Kaplan–Meier method. The 45-degree diagonal line indicates perfect calibration.

Figure S4. Kaplan-Meier curves comparing cancer-specific survival among patients with distant lymph node-only metastasis, other-sites-only metastasis, and combined distant lymph node plus other sites metastasis in the training cohort. P values were calculated using the log-rank test.

Figure S5. Three-month landmark Kaplan-Meier analysis of cancer-specific survival according to treatment modality. Cancer-specific survival curves stratified by treatment modality among patients with stage IVB1, IVB2, and IVB3 in the training cohort (A) and the internal validation cohort (B). Cancer-specific survival curves stratified by treatment modality among patients with stage IVB1 and IVB2 in the external validation cohort (C). Patients who died or were censored within 3 months after diagnosis were excluded, and survival time was recalculated from the 3-month landmark time point. P values were calculated using the log-rank test. Radio, radiotherapy; Chemo, chemotherapy.

Figure S6. Cancer-specific survival curves among patients who received no radiotherapy, primary site radiotherapy alone, or primary and metastatic sites radiotherapy in the external validation cohort. (A) Overall external validation cohort. (B) Stage IVB1 subgroup. (C) Stage IVB2 subgroup. P values were calculated using the log-rank test.

Figure S7. Cancer-specific survival curves stratified by immunotherapy in the external validation cohort. (A) Overall external validation cohort. (B) Stage IVB1 subgroups. (C) Stage IVB2 subgroups. P values were calculated using the log-rank test.

Figure S8. Cancer-specific survival curves stratified by primary site surgery in patients with stage IVB1, IVB2, IVB3 in the training cohort (A), the internal validation cohort (B). Cancer-specific survival curve stratified by treatment modality in patients with stage IVB1, IVB2 in the external validation cohort (C). P values were calculated using the log-rank test.

Figure S9. Three-month landmark Kaplan-Meier analysis of cancer-specific survival according to primary site surgery. Cancer-specific survival curves stratified by primary site surgery among patients with stage IVB1, IVB2, and IVB3 in the training cohort (A) and the internal validation cohort (B). Cancer-specific survival curves stratified by treatment modality among patients with stage IVB1 in the external validation cohort (C). Patients who died or were censored within 3 months after diagnosis were excluded, and survival time was recalculated from the 3-month landmark time point. P values were calculated using the log-rank test.

Figure S10. Cancer-specific survival curves stratified by distant metastatic site surgery in patients with stage IVB1, IVB2, IVB3 in the training cohort (A), the internal validation cohort (B). Cancer-specific survival curve stratified by treatment modality in patients with stage IVB1, IVB2 in the external validation cohort (C). P values were calculated using the log-rank test.

Figure S11. Three-month landmark Kaplan–Meier analysis of cancer-specific survival according to distant metastatic-site surgery. Cancer-specific survival curves stratified by distant metastatic-site surgery among patients with stage IVB1, IVB2, and IVB3 in the training cohort (A) and the internal validation cohort (B). Cancer-specific survival curves stratified by treatment modality among patients with stage IVB1 in the external validation cohort (C). Patients who died or were censored within 3 months after diagnosis were excluded, and survival time was recalculated from the 3-month landmark time point. P values were calculated using the log-rank test.
